# Artificial Intelligence Pipeline for Mammography-Based Breast Cancer Detection: An Integrated Systematic Review and Large-Scale Experimental Validation

**DOI:** 10.3390/medicina61122237

**Published:** 2025-12-18

**Authors:** Daniel Añez, Giuseppe Conti, Juan José Uriarte, José-Javier Serrano-Olmedo, Ricardo Martínez-Murillo, Oscar Casanova-Carvajal

**Affiliations:** 1 Escuela Superior de Ingeniería, Ciencia y Tecnología, Universidad Internacional de la Empresa UNIE, 28015 Madrid, Spain; 2 Departamento de Eléctrica, Electrónica, Automática y Física Aplicada, Escuela Técnica Superior de Ingeniería y Diseño Industrial ETSIDI, Universidad Politécnica de Madrid, 28040 Madrid, Spain; g.conti@upm.es; 3 Information Processing and Telecommunications Center (IP&T Center), Universidad Politécnica de Madrid, 28040 Madrid, Spain; 4 ARIES Research Group, Escuela Politécnica Superior, Universidad Antonio de Nebrija, 28015 Madrid, Spain; juriarte@nebrija.es; 5 Centro de Tecnología Biomédica, Campus de Montegancedo, Universidad Politécnica de Madrid, 28040 Madrid, Spain; josejavier.serrano@upm.es; 6 Departamento de Tecnología Fotónica y Bioingeniería, ETSI Telecomunicaciones, Universidad Politécnica de Madrid, 28040 Madrid, Spain; 7 Centro de Investigación Biomédica en Red para Bioingeniería, Biomateriales y Nanomedicina, Instituto de Salud Carlos III, 28029 Madrid, Spain; 8 Neurovascular Research Group, Department of Translational Neuroscience, Instituto Cajal, CSIC, 28002 Madrid, Spain; r.martinez@cajal.csic.es

**Keywords:** breast cancer, mammography, artificial intelligence, convolutional neural networks, classification models, support vector machines, deep learning, computer-assisted diagnostic systems, machine learning

## Abstract

*Background and Objectives:* Breast cancer remains a leading cause of cancer-related morbidity and mortality worldwide, and robust, interpretable artificial intelligence (AI) pipelines are increasingly being explored to support mammography-based detection. This study combines a PRISMA 2020-compliant systematic review with an original experimental validation to characterize current evidence and address identified gaps in reproducibility and interpretability. *Materials and Methods:* A PRISMA 2020-guided systematic review and an original experimental study were conducted. The review searched PubMed and Scopus/ScienceDirect for studies using convolutional neural networks (CNNs), support vector machines (SVMs) or eXtreme Gradient Boosting (XGBoost) for breast cancer detection in mammography and related imaging modalities, and identified 45 eligible articles. In parallel, we implemented and evaluated representative CNN (ResNet-50, EfficientNetB0 and MobileNetV3-Small) and classical machine learning (SVM and XGBoost) pipelines on the CBIS-DDSM dataset, following a CRISP-DM-inspired workflow and using Grad-CAM and SHAP to provide image- and feature-level explanations within a reproducible machine-learning-operations (MLOps)-oriented framework. *Results:* The systematic review revealed substantial heterogeneity in datasets, preprocessing pipelines, and validation strategies, with a predominant reliance on internal validation and limited use of explainable AI methods. In our experimental evaluation, ResNet-50 achieved the best performance (AUC-ROC 0.95; sensitivity 89%), followed by XGBoost (AUC-ROC 0.90; sensitivity 74%) and SVM (AUC-ROC 0.84; sensitivity 66%), while EfficientNetB0 and MobileNetV3-Small showed lower discrimination. Grad-CAM produced qualitatively plausible heatmaps centered on annotated lesions, and SHAP analyses indicated that simple global image-intensity and size descriptors dominated the predictions of the classical models. *Conclusions:* By integrating systematic evidence and large-scale experiments on CBIS-DDSM, this study highlights both the potential and the limitations of current AI pipelines for mammography-based breast cancer detection, underscoring the need for more standardized preprocessing, rigorous external validation, and routine use of explainable AI before clinical deployment.

## 1. Introduction

Breast cancer is the most common malignant disease among women worldwide and one of the leading causes of cancer-related mortality. According to the GLOBOCAN estimates, there were approximately 20 million new cancer cases and 9.7 million deaths worldwide in 2022, a number projected to reach 35 million by 2050 [1]. In Europe alone, breast cancer represented 29.4% of all new cancer diagnoses and 16.7% of cancer deaths among women during the same year [2]. Although the overall five-year survival rate exceeds 90% when the disease is detected in an early stage, it drops to between 50% and 80% for patients diagnosed in advanced stages. These statistics highlight the persistent need for reliable, scalable, and explainable technological innovations that can support early detection and reduce subjectivity in diagnostics in clinical settings.

Traditional diagnostic imaging modalities (mammography, ultrasound, and magnetic resonance imaging (MRI) [3]) remain the cornerstone of breast cancer screening and monitoring. However, their interpretation is partially subjective and highly dependent on the radiologist’s experience, leading to variability in sensitivity and specificity between healthcare institutions. In the last decade, the combination of medical imaging, large-scale datasets, and artificial intelligence (AI) has significantly reshaped the field of computer-aided diagnosis (CAD). AI systems, particularly those based on deep learning, have demonstrated notable success in automating lesion detection, segmentation, and classification tasks. These advances have improved diagnostic performance while reducing interpretation time, marking a paradigm shift in how imaging data are analyzed. However, important challenges remain regarding reproducibility, explainability, and clinical integration.

Convolutional neural networks (CNNs) [4] are among the most widely used deep-learning architectures in breast cancer imaging. They automatically learn hierarchical visual representations directly from pixel data, removing the need for manual feature engineering. CNNs have shown excellent performance in identifying subtle patterns in mammograms, ultrasound, and MRI scans. However, challenges persist, including limited interpretability, high data requirements, and the risk of overfitting when datasets are small or imbalanced. These constraints have motivated the continued use of classical machine-learning algorithms such as support vector machines (SVM) [5] and eXtreme Gradient Boosting (XGBoost) [6], which are based on carefully designed feature extraction pipelines. Although classical models require explicit feature definitions, for example, texture, shape, or radiomic descriptors, they often deliver competitive accuracy, particularly when data availability is limited, making their comparison with deep-learning approaches an ongoing and clinically relevant research topic.

Recent research has explored hybrid architectures that integrate CNN-based feature extraction with SVM or XGBoost classifiers, combining the representational power of deep learning with the robustness of classical models. In parallel, explainability has emerged as a central requirement in medical AI. Tools such as Gradient-weighted Class Activation Mapping (Grad-CAM) [7] for CNNs and SHapley Additive exPlanations (SHAP) [8] for tabular models enhance transparency by highlighting the image regions or variables driving the model’s decision-making process.

A comprehensive survey of Explainable Artificial Intelligence (XAI) techniques for medical imaging is presented in [9]. Various visualization methodologies, including perturbation-based, gradient-based, and attention-based approaches, are examined to evaluate their effectiveness in enhancing the interpretability and clinical relevance of deep learning models.

These XAI techniques are increasingly valued for supporting trust and facilitating clinical adoption, which motivated their inclusion in this study.

A key publicly available resource supporting the development and benchmarking of breast imaging algorithms is the Curated Breast Imaging Subset of the Digital Database for Mammography Screening (CBIS-DDSM), which contains approximately 1566 annotated mammographic studies with pixel-level segmentation masks and clinical metadata [10]. This dataset, together with advances in scalable computing and big data processing frameworks, has enabled reproducible experimentation across various AI architectures and parameter configurations.

Despite promising results reported in the literature, the field faces several unresolved challenges. The lack of standardized evaluation protocols and external validation limits the comparability of cross-study results. Moreover, conflicting findings persist regarding the superiority of deep-learning models over traditional algorithms: some studies argue that CNNs outperform SVM or XGBoost when trained on large datasets, whereas others describe comparable performance using handcrafted features and smaller samples. This divergence highlights the need for a more comprehensive synthesis of evidence to clarify current trends and identify best practices in the field.

Therefore, the present study adopts a two-part design that combines a systematic review with an experimental evaluation.In the first part, we conduct a PRISMA 2020-based systematic review of AI-driven breast cancer detection methods across imaging modalities, with particular emphasis on the comparative performance of CNN, SVM, and XGBoost algorithms. Following the PRISMA 2020 methodology [11], this review synthesized the findings of 45 peer-reviewed studies retrieved from PubMed and Scopus.

In the second part, informed by the gaps and trends identified in the review, we develop and assess a scalable and reproducible pipeline for breast cancer detection using the CBIS-DDSM [10] dataset. This pipeline integrates data standardization, image preprocessing, model training, and explainability analysis for both deep-learning and classical machine-learning models, with Grad-CAM and SHAP providing complementary insights into model behavior. To our knowledge, no previous study has jointly combined a systematic review of CNN, SVM, and XGBoost based approaches with an experimental pipeline implementation that emphasizes methodological transparency and XAI practices. We anticipate that this integrated perspective will clarify the complementary strengths of these model families, highlight the need for rigorous patient-level validation, and emphasize the growing role of explainable AI for clinically reliable breast cancer detection.

Overall, this work combines:

(i) A PRISMA-2020-based systematic review of convolutional and classical machine-learning approaches for mammographic detection of breast cancer.
(ii) An experimental comparison of CNN-, SVM-, and XGBoost-based pipelines on the CBIS-DDSM [10] dataset with Grad-CAM and SHAP interpretability.
(iii) An implementation of a reproducible MLOps-oriented workflow for model training, tracking, and deployment.

## 2. Materials and Methods

### 2.1. Protocol and Reporting Guideline

This systematic review was conducted and reported according to the Statement of Preferred Reporting Items for Systematic Reviews and meta-analysis (PRISMA) 2020 [11]. The review protocol was prospectively registered on the International Prospective Register of Systematic Reviews (PROSPERO) under registration number CRD420251141054.

The purpose of this review is to provide a reproducible synthesis of the current scientific evidence on artificial-intelligence (AI) methods applied to breast cancer detection using medical-imaging data. Specifically, the review identifies, evaluates, and compares studies employing convolutional neural networks (CNNs), support vector machines (SVM), and eXtreme Gradient Boosting (XGBoost) algorithms for the diagnosis or classification of breast lesions across mammography, ultrasound, magnetic resonance imaging (MRI), and multimodal imaging pipelines. The claim of this work is that, by applying a standardized PRISMA 2020 protocol, it is possible to objectively assess methodological rigor, diagnostic performance, and explainability practices across AI-driven approaches to breast-cancer detection. This claim will be further refined after the final validation of data extraction and bias assessment.

All materials, extracted data, and analysis protocols associated with this review will be made publicly available after publication to ensure reproducibility and transparency. The imaging data used in this work were obtained from the Curated Breast Imaging Subset of the Digital Database for Mammography Screening (CBIS-DDSM), which is publicly hosted by The Cancer Imaging Archive (TCIA). According to TCIA’s data-use policy, the CBIS-DDSM [10] collection is freely available for non-commercial research and educational purposes and is distributed under a Creative Commons Attribution 3.0 Unported (CC BY 3.0) license. The dataset complies with the Health Insurance Portability and Accountability Act (HIPAA) regulations and has been de-identified according to the TCIA’s data curation and patient-privacy protocols, as stated on its official repository website (https://www.cancerimagingarchive.net/collection/cbis-ddsm/, accessed on 4 December 2025).

No new human or animal data was collected for this review. Therefore, no institutional ethical approval or informed consent was required. The authors declare that no confidential or proprietary datasets were used.

Generative artificial-intelligence (GenAI) tools were used solely for linguistic editing and improvement of grammar, clarity, and formatting. No GenAI systems were used to generate data, results, or interpretations. The authors remain fully responsible for the accuracy, validity, and integrity of the content presented in this manuscript.

### 2.2. Information Sources and Search Strategy

The literature search was conducted to identify peer-reviewed studies that applied artificial-intelligence (AI) methods for the detection or diagnosis of breast cancer using medical-imaging data. Two major bibliographic databases were consulted: PubMed (U.S. National Library of Medicine) and Scopus (Elsevier). The search included all records available until 15 June 2024 and was restricted to studies published within the preceding three years.

The PubMed query combined controlled vocabulary and free-text terms related to breast cancer, imaging modalities, and AI techniques, as shown below:

(“breast cancer” OR “breast neoplasm”) AND (“deep learning” OR “CNN” OR “convolutional neural network” OR “machine learning” OR “support vector machine” OR “XGBoost”) AND (“mammography” OR “ultrasound” OR “magnetic resonance imaging” OR “MRI” OR “medical imaging”)

The Scopus (ScienceDirect, specifically) query also included terms aligned with the purpose of our research process, combining different AI techniques focusing on breast cancer detection, as shown below:

convolutional neural networks; breast cancer; detection; machine learning; mammograms; deep learning; xgboost; support vector machines

The search retrieved a total of 645 records from PubMed and 115 records from ScienceDirect, yielding 680 unique records before duplicate removal. All retrieved records were exported in CSV format and imported into a screening tool for systematic deduplication and subsequent eligibility assessment.

**Figure 1:**
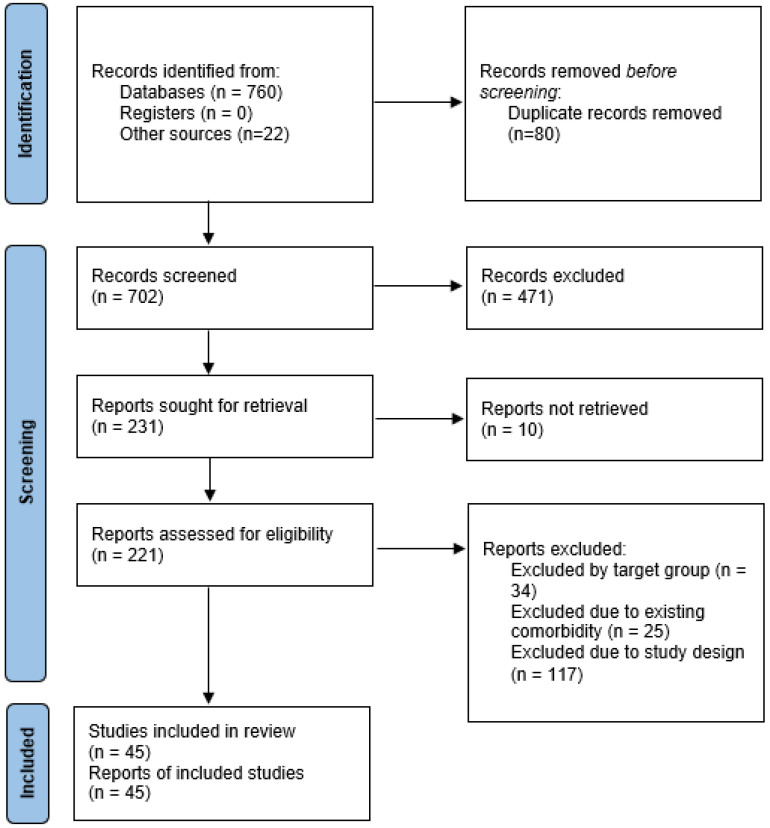
PRISMA 2020 flow diagram showing the identification, screening, eligibility, and inclusion stages of the systematic review.

The search process adhered to the principles of FAIR data-management (Findable, Accessible, Interoperable, and Reusable) and followed the recommendations of PRISMA 2020 for systematic literature identification [11]. The eligibility criteria applied during the screening and selection stages are detailed in Section 2.3, and the general identification process is illustrated in the PRISMA 2020 flow diagram (Figure 1).

### 2.3. Eligibility Criteria

The eligibility criteria were defined prior to the screening process to ensure consistency and reproducibility. The review focused on peer-reviewed studies that applied artificial-intelligence (AI) methods for breast-cancer detection using medical-imaging data. The inclusion and exclusion criteria were established according to the objectives of this review and aligned with the PRISMA 2020 framework [11].

Inclusion criteria:

(i) Articles published within the previous three years (2021–2025) to ensure the review captured the most recent developments in AI-based diagnostic modeling.
(ii) Studies performed on human subjects or datasets derived from human breast-imaging examinations.
(iii) Studies focused on breast-cancer detection, classification, or diagnostic support.
(iv) Studies based on mammographic images or multimodal imaging pipelines including mammography as a primary source.
(v) Research employing AI techniques, specifically convolutional neural networks (CNNs), support vector machines (SVM), or eXtreme Gradient Boosting (XGBoost).
(vi) Publications available as full-text peer-reviewed journal or conference papers with a valid digital object identifier (DOI).

Exclusion criteria:

(i) Studies using non-human samples, synthetic phantoms, or simulated data without clinical validation.
(ii) Research addressing cancer types other than breast cancer or tasks unrelated to detection (e.g., prognosis or treatment planning).
(iii) Articles not focused on imaging-based detection or that lacked quantitative diagnostic evaluation.
(iv) Preprints, editorials, reviews, theses, or unpublished manuscripts.
(v) Papers lacking a DOI or not retrievable in full text.
(vi) Studies whose objectives, imaging pipelines, and outcome measures were not compatible with the research questions and analytical framework adopted in this study.

These criteria were chosen to ensure that only original, methodologically comparable studies were included. Restricting the search to the most recent four-year period allowed the synthesis of up-to-date methodological advances while avoiding redundancy from earlier systematic reviews. Focusing on human mammography datasets guaranteed clinical relevance and consistency in imaging modality, while requiring the use of CNN, SVM, or XGBoost models ensured methodological comparability among studies. The inclusion and exclusion criteria were independently applied during title/abstract and full-text screening by the review authors, and disagreements were resolved through discussion until consensus was reached. Additional details on the PRISMA-2020 main checklist and abstract are provided in the Supplementary Materials (Tables S1 and S2, respectively).

### 2.4. Study Selection Process

The study selection process was performed according to the PRISMA 2020 statement [11]. All records identified from PubMed and Scopus (ScienceDirect) were exported and merged into a single reference database. Duplicate entries were automatically detected and removed using the built-in deduplication features of the reference-management software, followed by a manual verification step to ensure data integrity.

After removal of 80 duplicates, 702 unique records were retained for title and abstract screening. Three reviewers independently screened all records to determine their potential eligibility according to the predefined inclusion and exclusion criteria described in Section 2.3. Disagreements were resolved through discussion until consensus was reached. Studies that could not be clearly excluded during abstract screening proceeded to full-text evaluation.

A total of 221 full-text articles were assessed for eligibility. Of these, 176 were excluded for the following reasons:

(i) Non-breast-cancer focus or irrelevant target population (n=34);
(ii) Comorbidity or overlapping clinical conditions that precluded specific breast-cancer analysis (n=25); and
(iii) Unsuitable study design, such as absence of quantitative diagnostic evaluation or insufficient methodological detail (n=117).

Ultimately, 45 studies met all eligibility criteria and were included in the qualitative synthesis. The general selection process, including identification, selection, eligibility assessment, and final inclusion, is summarized in the PRISMA 2020 flow diagram (Figure 1). This transparent selection procedure ensures that the included literature represents the most methodologically robust and clinically relevant research on AI-based breast-cancer detection.

### 2.5. Data Extraction and Management

Following the Cross-Industry Standard Process for Data Mining (CRISP-DM) model [12], the data-extraction and preparation stages of this study were designed to ensure a transparent and reproducible workflow. The dataset used was the Curated Breast Imaging Subset of the Digital Mammography Screening Database (CBIS-DDSM) [10], publicly available through The Cancer Imaging Archive (TCIA). All image files and metadata were obtained in DICOM format and processed to build a clean and structured dataset suitable for deep-learning and machine-learning experimentation.

The extraction process involved parsing the CSV metadata accompanying the images to generate a unified table containing patient identifiers, lesion type, laterality, view position, breast density, and pathological labels. Each entry was verified to confirm the one-to-one correspondence between the image and metadata records, ensuring that each DICOM study included a valid annotation mask and diagnostic label. Duplicate and corrupted files were not identified.

For efficient visualization and manual quality control, the DICOM images from the CBIS-DDSM [10] collection were converted to 16-bit PNG format. This conversion enabled faster inspection of annotation consistency and textual artifacts while reducing the computational burden of DICOM decoding during training. By using 16-bit PNG, we retained the original dynamic range and image fidelity required for downstream processing.

Associated metadata were stored in tabular format (CSV and Parquet) for programmatic access within the modeling pipeline. Integrity checks confirmed the internal consistency of the dataset, including the verification of file counts, label balance, and laterality distribution. Statistical summaries of these parameters are presented in Section 2.6. Additional validation ensured proper alignment between image dimensions and ROI masks in all samples.

All data-handling and validation processes were implemented in Python (version 3.11.9) using the pydicom, pandas, and numpy libraries, following the “Data Understanding” and “Data Preparation” phases of the CRISP-DM framework. Scripts were version-controlled to ensure reproducibility and traceability. The complete codebase used for parsing, conversion, and validation will be made publicly available in the project’s open-access GitHub (version 2.51.0.windows.2) repository upon publication, along with documentation describing the data schema and processing pipeline. This repository is already available and with open access in GitHub (https://github.com/danielanezd/breast_cancer_detection, accessed on 8 December 2025)

### 2.6. Risk of Bias Assessment

The risk of bias analysis was carried out on two complementary levels:

(i) An internal evaluation of the CBIS-DDSM [10] dataset used for experimentation;
(ii) A qualitative evaluation of methodological bias within the studies included in the systematic review.

#### 2.6.1. Dataset-Level Bias Assessment

Before model training, the CBIS-DDSM dataset [10] was analyzed to identify potential sources of bias that could affect model learning. Several quantitative checks were performed to verify the balance of the class, the distribution of laterality, and the presence of textual artifacts in the images.

**Figure 2:**
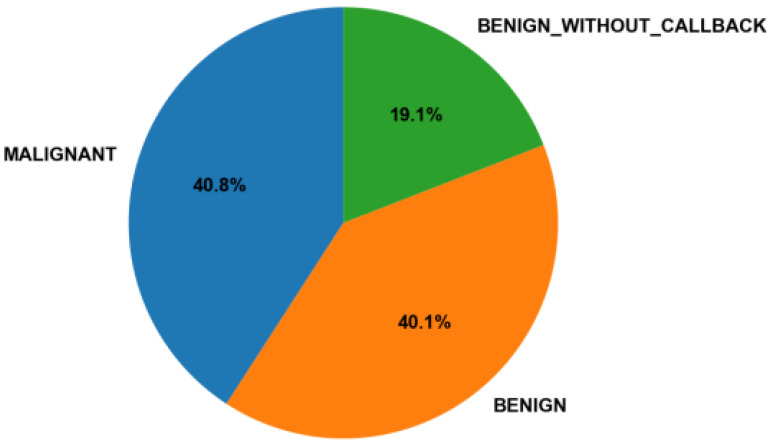
Class distribution in the CBIS-DDSM dataset [10]. As shown in Figure 2, the dataset includes three categories: Malignant. (confirmed cancer), Benign (confirmed benign anomaly), and Benign Without Callback (no further studies required).

**Figure 3:**
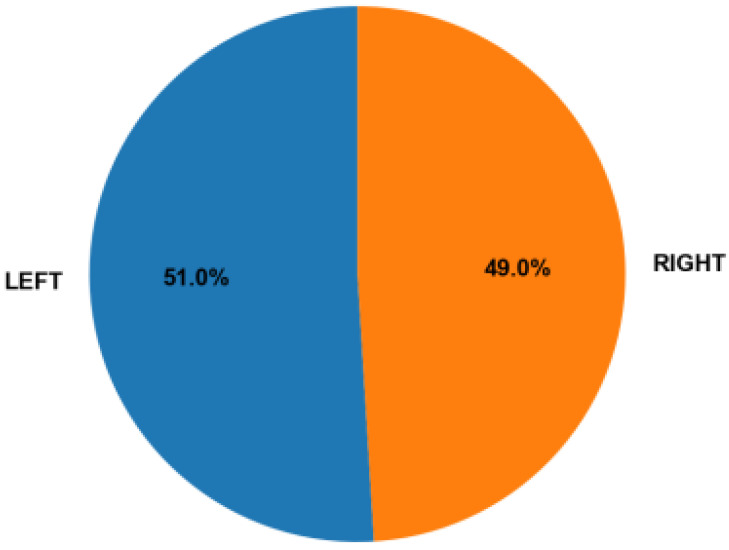
Laterality distribution in the CBIS-DDSM dataset [10]. As shown in Figure 3, the dataset includes a comparable number of left- and right-breast images, indicating no notable lateral predominance.

**Figure 4:**
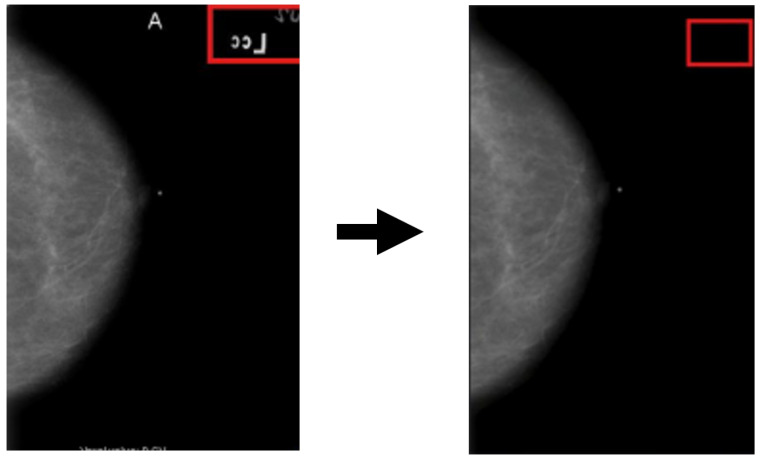
Detection and masking of textual and annotation artifacts using OCR. As shown in the figure, alphabetical characters were automatically identified and masked to prevent non-anatomical information from biasing the learning process. From left to right: red box shows one of the place in which artifacts were detected and then it shows the same place in which the artifacts were masked.

- **Balance of classes:** The proportions of benign and malignant cases were calculated and compared to ensure that both categories were adequately represented (Figure 2). The dataset showed a nearly balanced distribution, reducing the risk of the model overfitting toward one diagnostic class.

- **Laterality distribution:** The number of images of the left and right breasts was examined to detect potential spatial bias (Figure 3). The frequencies were similar, indicating that there was no significant lateral predominance.

- **Textual and annotation artifacts:** A complementary analysis using optical-character recognition (OCR) was conducted to identify alphanumeric characters and embedded labels within mammograms. Figure 4 shows representative examples in which text elements could confound pixel-based learning. These regions were masked prior to model training to prevent non-anatomical features from influencing predictions.

- **Consistency of the metadata:** The image identifiers, the view positions, and the lesion labels were cross-checked to verify the integrity of the data set and to avoid duplication or misalignment between the images and the annotations.

These quantitative and qualitative inspections confirmed that CBIS-DDSM [10] provides a reliable basis for AI research but still exhibits minor risks of bias arising from textual artifacts and slight variations in pathological representation.

#### 2.6.2. Methodological Bias Between the Reviewed Studies

In addition to the analysis of the dataset, each of the 45 included articles was examined to identify broader methodological biases. The assessment focused on five main domains relevant to AI-based medical-imaging research:

(i) Data bias: imbalance in class composition or lack of demographic diversity in training datasets;
(ii) Validation bias: absence of external validation, with reliance on internal train–test splits;
(iii) Reporting bias: incomplete disclosure of metrics or experimental settings;
(iv) Reproducibility bias: lack of code, data, or detailed implementation information; and
(v) Explainability bias: omission or superficial use of interpretability methods such as Grad-CAM or SHAP.

The evaluation was performed by the first author through manual review of the methodology and results sections of each publication. Ambiguous cases were re-checked for consistency. No numerical scores were assigned; instead, qualitative judgments of low, moderate, or high bias were recorded for each domain.

The results of both dataset-level and study-level assessments are discussed in Section 3, together with their implications for model generalizability and reliability in clinical practice.

### 2.7. Synthesis Methods

This study evaluated two families of models applied to the CBIS-DDSM dataset [10]: convolutional neural network (CNN) architectures for image-based classification, and traditional machine-learning algorithms trained on handcrafted statistical features derived from the same images. Each model family followed an independent experimental workflow, consistent with its methodological requirements.

#### 2.7.1. Convolutional Neural Networks

Three CNN architectures were trained and evaluated: ResNet-50 [13], EfficientNetB0 [14], and MobileNetV3-Small [15]. The models were implemented using the PyTorch (version 2.8.0+cu129 that includes CUDA support) framework with transfer learning from ImageNet weights. The dataset was partitioned into training subsets (64%), validation subsets (16%) and test subsets (20%) using stratified sampling to maintain the class and laterality balance described in Section 2.6. The validation subset was used during training to monitor performance metrics and apply early stopping, preventing overfitting. All networks were optimized using the Adam optimizer and categorical cross-entropy loss. Training was performed using mini-batch gradient descent, and convergence was determined by stabilizing the validation loss.

Performance was quantified in the independent test set using the area under the receiver operating characteristic curve (AUC-ROC), accuracy, precision, recall (sensitivity), F1-score, and specificity. These metrics were selected to ensure comparability across architectures and to provide a balanced assessment of model performance from both clinical and computational perspectives.

#### 2.7.2. Machine-Learning Models

In parallel, two classical machine-learning algorithms were implemented: Support Vector Machine (SVM) [16] and eXtreme Gradient Boosting (XGBoost) [6]. These models were trained on handcrafted features extracted directly from full mammographic images, including mean intensity, standard deviation, width, and height. These descriptors provided a simple yet interpretable representation of the image characteristics without involving convolutional processing. For the SVM and XGBoost experiments, the dataset was divided into 80% for training and 20% for testing. Hyperparameters such as the kernel type and regularization parameter for SVM, and tree depth and learning rate for XGBoost, were optimized using internal cross-validation within the training set. The performance of the model was evaluated on the held test set using the same metrics applied to CNN models (AUC-ROC, accuracy, precision, recall, F1-score, and specificity), ensuring a consistent basis for comparison.

#### 2.7.3. Explainability and Interpretation

To interpret the predictions of the model, Gradient-weighted Class Activation Mapping (Grad-CAM) was applied to CNN output to visualize the salient image regions associated with each decision [7]. For machine-learning models, SHAP values (SHapley Additive exPlanations) were used to quantify the contribution of each hand-made feature to the prediction result [8]. These analyses allowed a qualitative assessment of whether the models based their decisions on clinically meaningful information.

All experiments were conducted in Python, using the PyTorch and scikit-learn libraries for the deep-learning and machine-learning pipelines, respectively. The synthesis of the results was performed by aggregating quantitative metrics and comparing them between model families, as presented in Section 3. As no external dataset was used for validation, the reported findings should be interpreted within the scope of internal evaluation, acknowledging that future studies may require cross-dataset and multi-institutional verification to assess generalizability.

### 2.8. Data Preprocessing and Augmentation

Preprocessing and augmentation were applied to improve the robustness and generalization of convolutional neural network (CNN) models while preserving the diagnostic quality of the mammographic data. All transformations were applied only to the training subset. The validation and test sets remained unchanged to provide an unbiased evaluation.

#### 2.8.1. Preprocessing

Each DICOM image was converted to a floating-point tensor and normalized so that the pixel intensities ranged between 0 and 1. Because the CNN architectures used in this study were pre-trained on ImageNet, the grayscale mammograms were replicated into three identical channels to match the expected RGB input. All images were then resized to 224 × 224 pixels to maintain a consistent input dimension for the ResNet-50, EfficientNetB0 and MobileNetV3-Small networks. This resolution was chosen as a pragmatic compromise between computational feasibility and preservation of lesion morphology; however, such downsampling inevitably reduces the original pixel resolution and may partially smooth very small structures such as isolated microcalcification clusters. Consequently, our experimental results should be interpreted primarily at the lesion- and breast-level classification scale rather than as an optimized solution for dedicated microcalcification detection.

#### 2.8.2. Augmentation

To reduce overfitting and simulate realistic variability in image acquisition, we applied a standard set of data-augmentation methods during training—namely random rotations, horizontal and vertical flips, Gaussian blur, adjustments to brightness and contrast, and random cropping or padding. We then used an automated hyperparameter-tuning framework to search across a predefined range of settings (e.g., learning rate, dropout, augmentation magnitude) and selected the configuration that provided the best performance. In addition, the training process incorporated a learning-rate scheduler and an early-stopping mechanism, helping the model converge appropriately while avoiding over-fitting.

### 2.9. Hardware and Software

#### 2.9.1. Hardware

All experiments were conducted on a dedicated workstation equipped with an AMD Ryzen 9950X3D CPU, 96 GB of RAM, and an NVIDIA RTX 5090 GPU with 32 GB of VRAM. The deep learning models were implemented in Python using PyTorch and trained on the GPU in a CUDA-enabled environment, while the classical machine learning models (XGBoost and SVM) were trained on the same machine using CPU-based computation.

#### 2.9.2. Software

During the preparation of this manuscript, an AI-based language tool (M365 Copilot, version bizchat.20251208.59.2) was utilized for the sole purpose of proofreading to improve grammar, spelling, and clarity. This tool was not used for any part of the core research process, such as literature search, data collection, analysis, or the generation of scientific insights. The authors hold full responsibility for the content and have reviewed all final text to verify its accuracy and ensure that the intended scientific meaning was maintained.

In the context of the PRISMA-2020 screening process, the Rayyan.ai web application was employed (version as of 3 December 2025 Product Update). This application offers features that facilitate the importation of article metadata, the full text, inclusion and exclusion criteria, and the voting process to resolve disagreements during the article screening process.

## 3. Results

This section presents the experimental results produced by the models developed in this study. It begins with an overview of the software architecture implemented to integrate data preprocessing, augmentation, model training, and evaluation. The subsequent subsections report the quantitative performance metrics of the convolutional neural network (CNN) models and the traditional machine-learning algorithms, followed by the qualitative analysis of model explainability through Grad-CAM and SHAP visualizations. Together, these results provide a comprehensive view of the system’s performance and interpretability in different modeling approaches.

### 3.1. Overview of Results and System Architecture

The software architecture developed for this study was designed to support the entire experimental workflow, from data preprocessing to model evaluation and explainability. The implementation followed a modular structure aligned with the CRISP-DM framework [12], ensuring that each phase of the process—data preparation, modeling, evaluation, and deployment—could be executed independently and reproduced consistently.

The core of the system is characterized by a controller module that orchestrates the different components responsible for data handling, model selection, and evaluation. The data-management layer handles the loading of DICOM images, preprocessing, and augmentation. The modeling layer encapsulates the convolutional neural network (CNN) architectures ResNet-50, EfficientNetB0, MobileNetV3-Small, the machine-learning models Support Vector Machine (SVM) and XGBoost—through a unified interface implemented using the Strategy Pattern. This design allows models to be interchanged without altering the training or evaluation logic. The evaluation layer computes quantitative performance metrics and triggers the explainability routines based on the Grad-CAM and SHAP methods.

**Figure 5:**
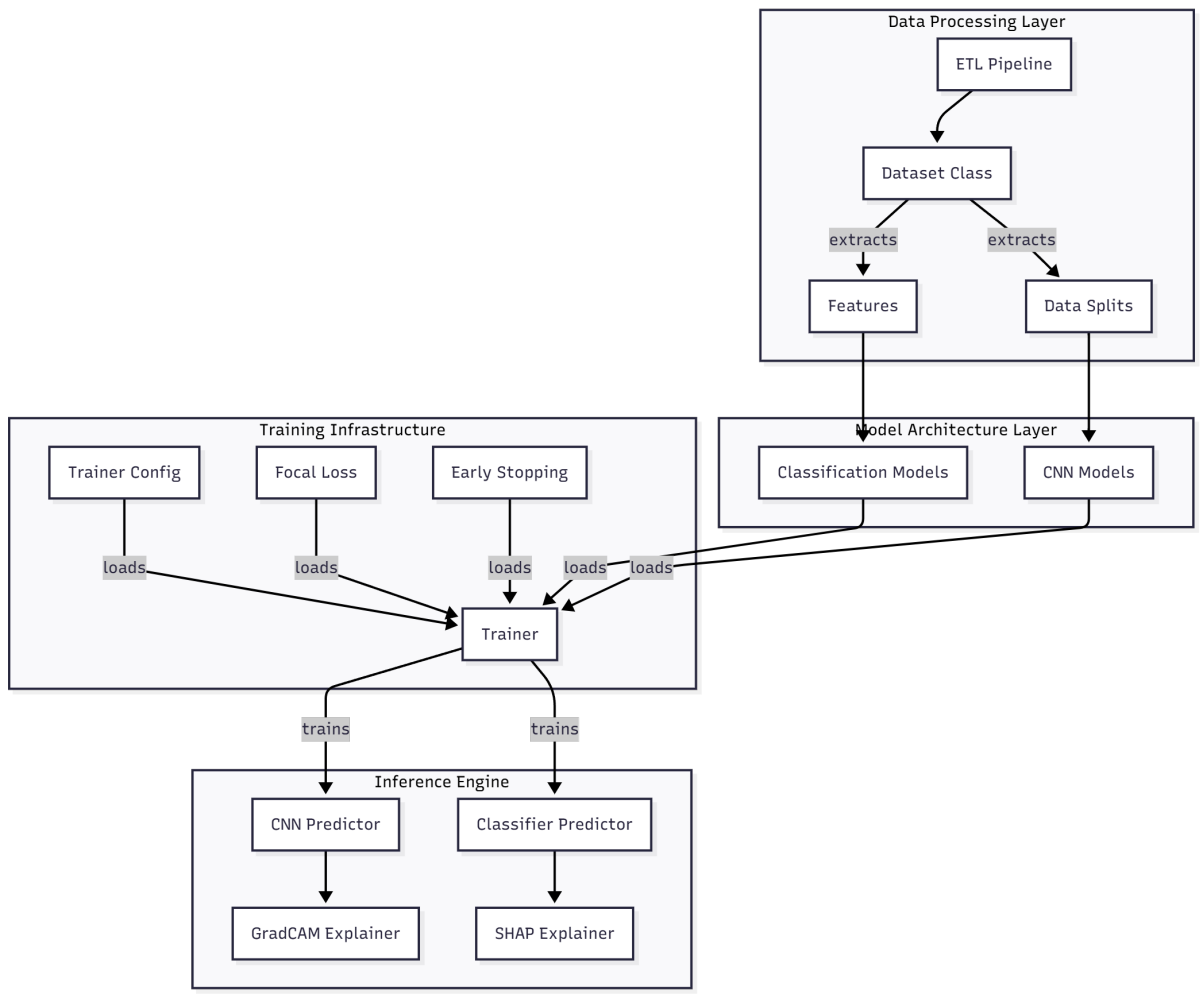
Software architecture and experimental workflow showing modular components for data preprocessing, augmentation, model training, evaluation, and explainability. Original figure created by the authors.

Figure 5 illustrates the overall structure of the software and the interaction between modules during the experimental workflow. The modularity of the design facilitates scalability, enabling future integration of additional datasets or architectures without major structural modifications.

### 3.2. Experimental Setup and Hyperparameter Optimization

All machine learning models evaluated in this study were trained using a unified experimental pipeline based on MLflow [17] and Optuna [18], ensuring methodological consistency, reproducibility, and systematic exploration of the hyperparameter space in all architectures (ResNet-50, EfficientNetB0, MobileNetV3-Small, SVM, and XGBoost).

MLflow was used to track each stage of the experiments, including parameter logging, metric storage, artifact management, and version control of all models. Each hyperparameter search was organized as a *parent run* representing a study, inside which Optuna executed multiple *nested trials*, each corresponding to a different hyperparameter configuration.

Hyperparameter optimization was performed using the Optuna Bayesian sampler (TPESampler) together with a MedianPruner to terminate underperforming trials early. For each trial, Optuna sampled a candidate set of hyperparameters, triggered a complete training cycle, and evaluated the model in a validation subset. The optimization objective for all models was malignant recall, due to its clinical relevance in minimizing false negatives during breast cancer screening.

This integrated MLflow–Optuna workflow provided systematic and efficient search capabilities, outperforming manual tuning, and enabling transparent comparison of thousands of logged metrics. The best-performing model of each study was automatically stored and versioned in the MLflow Model Registry for subsequent evaluation and deployment.

### 3.3. CNN Model Performance

**Figure 6:**
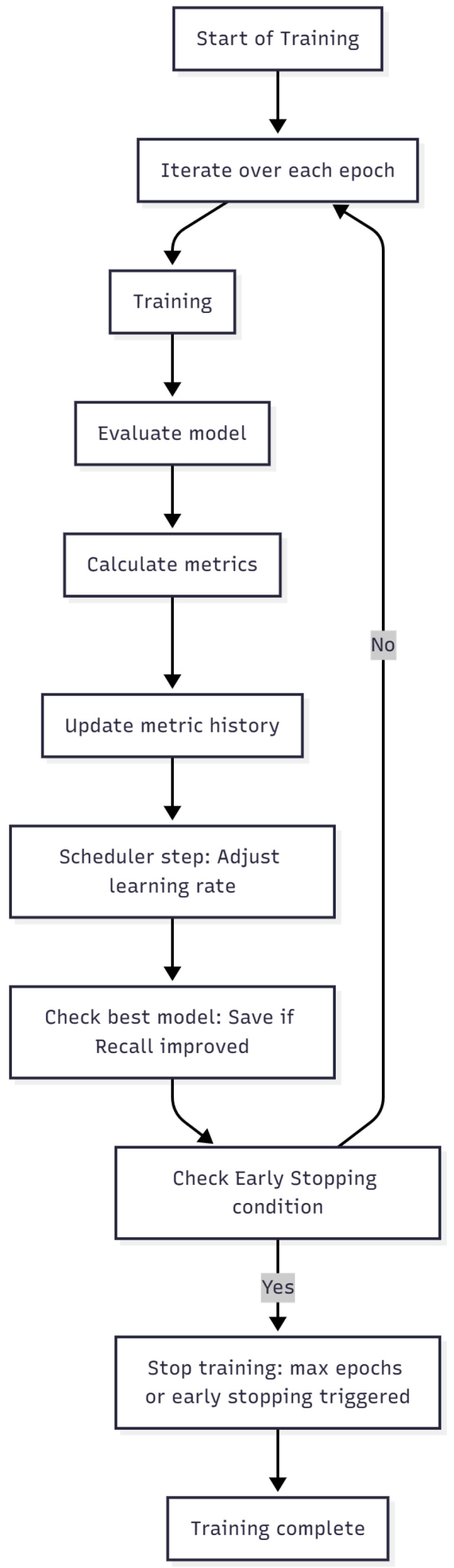
Training and validation loop used for all CNN architectures. Each epoch included data loading, augmentation, forward propagation, loss computation, backpropagation, and monitoring on the validation subset. Original figure created by the authors.

The convolutional neural network (CNN) models—ResNet-50, EfficientNetB0, and MobileNetV3-Small were trained using the 64/16/20 train–validation–test split described in Section 2.7. Each network followed the same training and validation loop, which controlled the learning rate, monitored validation loss, and applied early stopping once the convergence criteria were reached. Figure 6 shows the workflow implemented uniformly for all architectures.

#### 3.3.1. Training Configuration for CNN Models

The hyperparameter configurations summarized in Table 1 correspond to the results of the automated Optuna-based tuning stage integrated into our training pipeline. For each convolutional architecture (ResNet-50, EfficientNetB0, and MobileNetV3-Small), Optuna explored alternative settings for the backbone and classifier-head learning rates, weight decay, Focal Loss parameters (gamma and class weights α), mini-batch size, and early stopping patience, using malignant-class recall on the validation set as the optimization target. The values reported in Table 1 therefore represent the final hyperparameter selections yielded by this optimization process and were subsequently used in all experiments presented in the Results section.

#### 3.3.2. Evaluation Metrics for CNN Models

Model performance was measured in the independent test subset using the area under the receiver operating characteristic curve (AUC-ROC) and Recall (Sensitivity). These metrics capture both discriminative ability and clinical relevance for early cancer detection. Table 2 reports the quantitative results obtained for each CNN.

**Figure 7:**
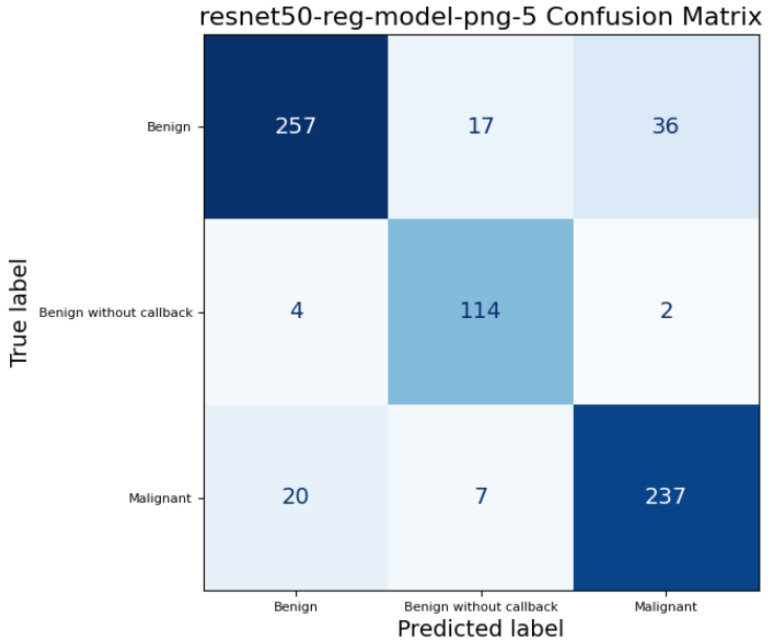
ResNet-50 Confusion Matrix on full mammograms samples.

**Figure 8:**
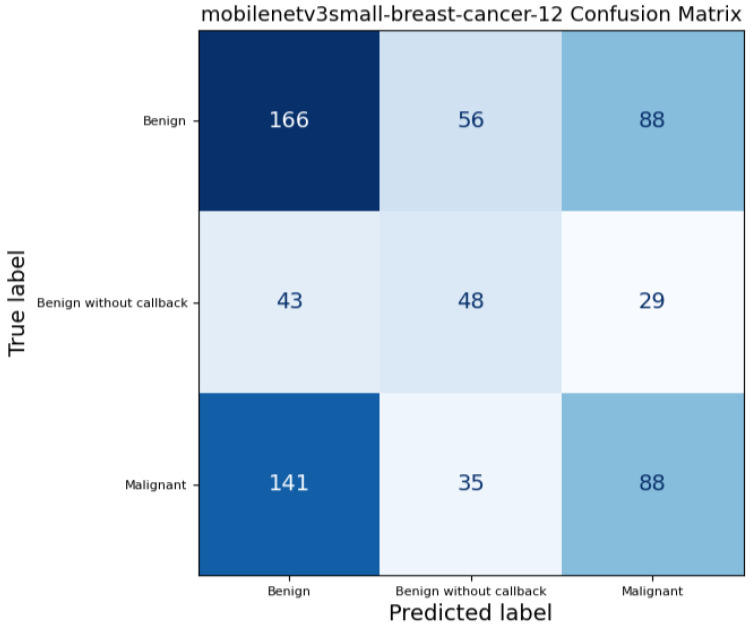
MobileNetV3-Small Confusion Matrix on full mammograms samples.

**Figure 9:**
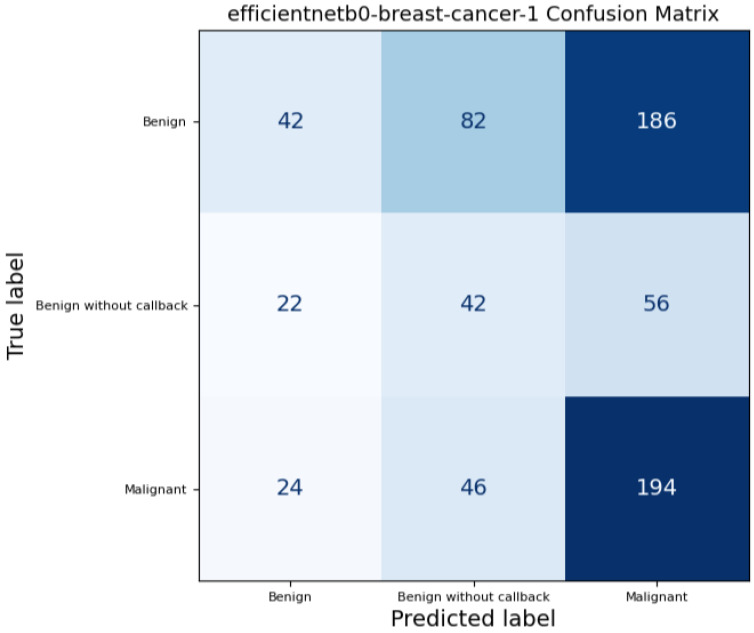
EfficientNetB0 Confusion Matrix on full mammograms samples.

In addition to calculating standard performance metrics for each model, we produced a confusion matrix to examine how each model classified the three target classes, these confusion matrices are represented by the Figure 7, Figure 8 and Figure 9. A confusion matrix is a table comparing the true class of each case with the class predicted by the model: entries along the diagonal represent correct predictions, while off-diagonal entries correspond to misclassifications. For example, a “true positive” (TP) indicates a case correctly identified as belonging to the positive class, whereas a “false negative” (FN) denotes a positive case wrongly classified as negative; similarly, “false positives” (FP) and “true negatives” (TN) describe incorrect and correct negative predictions, respectively. By analyzing these counts, we can see not only overall accuracy but also whether the model systematically misclassifies certain classes — for instance, consistently confusing benign and malignant lesions — which is critical when assessing clinical utility and reliability.

### 3.4. Machine Learning Model Performance

**Figure 10:**
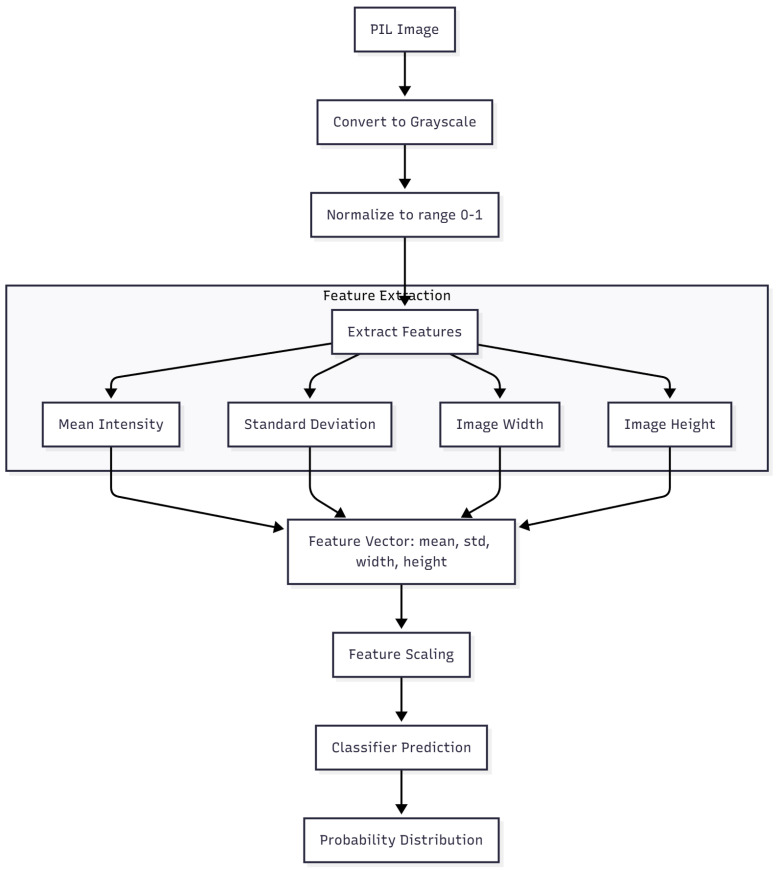
Workflow of the handcrafted feature extraction and classification process used for the SVM and XGBoost models. Original figure created by the authors.

In addition to the convolutional models, two classical machine-learning algorithms were trained and evaluated: Support Vector Machine (SVM) and eXtreme Gradient Boosting (XGBoost). Both models used the handcrafted features extracted directly from mammographic images, as described in Section 2.7. These features included mean intensity, standard deviation, image width, and image height, normalized to the range [0, 1]. The complete feature extraction and classification workflow is illustrated in Figure 10.

#### 3.4.1. Training Configuration for Machine Learning Models

In addition to the convolutional neural network models, we optimized the hyperparameters of two classical machine learning classifiers, namely XGBoost and support vector machines (SVM). Hyperparameter search was performed using the same Optuna-based optimization stage integrated into our pipeline, with malignant-class recall on the validation data as the objective function. For XGBoost, the search space included learning rate, tree depth and structure, regularization coefficients, and the number of boosting iterations, whereas for SVM it comprised the penalty parameter *C*, kernel type, gamma scheme, and class-weighting strategy. The resulting best configurations obtained after optimization were subsequently fixed and used in all reported experiments. The final tuned hyperparameters for XGBoost and SVM are summarized in Table 3 and Table 4, respectively.

#### 3.4.2. Evaluation Metrics for Machine Learning Models

Among classical ML approaches, XGBoost achieved the highest performance, indicating a strong ability to correctly identify malignant cases using simple statistical features, while SVM yielded lower but competitive results, as showing in Table 5, showing moderate performance but below both the XGBoost and the best CNN models. These results confirm that even minimal statistical descriptors extracted from mammographic images can support effective classification, although deep-learning architectures remain advantageous for feature learning and spatial context analysis.

**Figure 11:**
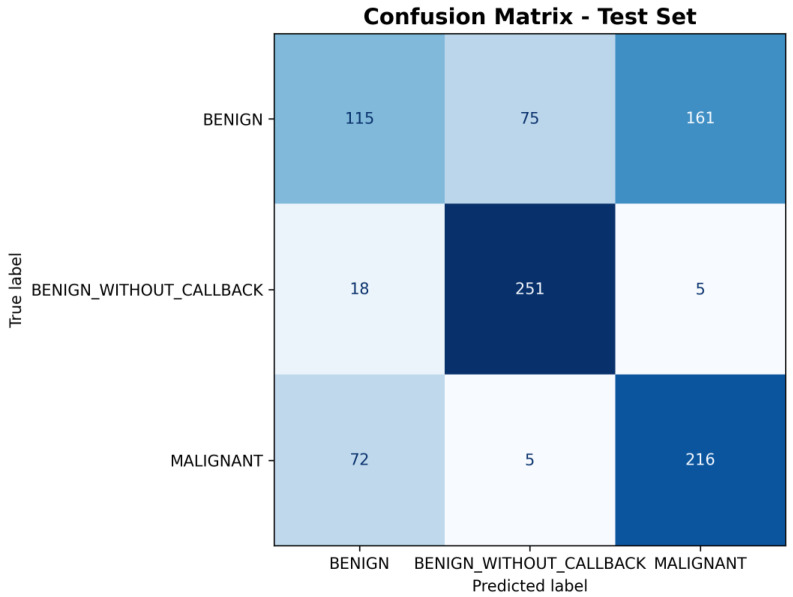
SVM Confusion Matrix on CBIS-DDSM [10] handcrafted features.

**Figure 12:**
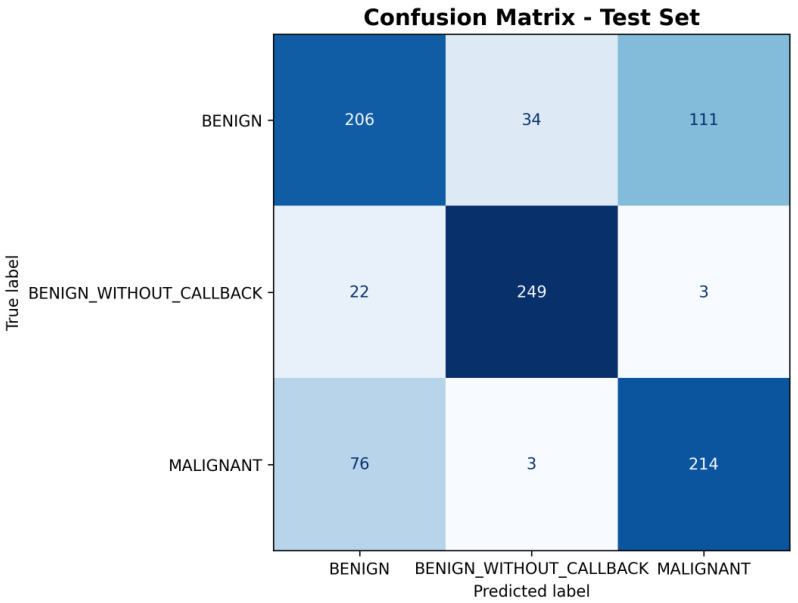
XGBoost Confusion Matrix on CBIS-DDSM [10] handcrafted features.

As with the CNN models, the performance of the classical classifiers is illustrated in the confusion matrices shown in Figure 11 and Figure 12, corresponding to the SVM and XGBoost models, respectively.

### 3.5. Explainability Results

To contextualize our approach within the broader landscape of medical XAI techniques reviewed by Bhati et al. [9], we performed explainability analyses to ensure model transparency and evaluate whether decisions were based on clinically meaningful features. Two complementary techniques representing the gradient-based and feature-attribution categories were applied: Gradient-weighted Class Activation Mapping (Grad-CAM) for the convolutional neural networks and SHapley Additive exPlanations (SHAP) for the classical machine-learning models.

#### 3.5.1. Grad-CAM Visualization

**Figure 13:**
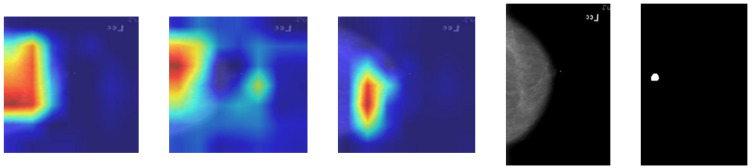
Grad-CAM visualizations for Patient 00038’s mammograms. Warmer colours (red/yellow) indicate regions that contributed most to the predicted malignant class, while cooler colours indicate low relevance. From **left** to **right**: MobileNetV3-Small, EfficientNetB0, and ResNet-50. The reference mammogram and the expert-annotated ROI are shown for comparison.

For CNN models, Grad-CAM [7] heatmaps were generated to highlight the image regions that contributed the most to each classification decision. The resulting activations showed that the networks focused primarily on the areas corresponding to the annotated lesions in the CBIS-DDSM dataset [10], confirming that the models’ decision processes were aligned with clinically relevant information These visualizations provide qualitative insight into model attention and feature contributions. Representative examples of each CNN architecture are shown in Figure 13.

Grad-CAM heatmaps use a colour scale in which warmer colours (red/yellow) correspond to a stronger positive contribution to the malignant prediction.

Among the three architectures, ResNet-50 produced the most coherent activation maps, with focus aligned to the lesion boundaries and minimal attention to irrelevant regions. EfficientNetB0 displayed a similar spatial focus but slightly more diffuse activation around the lesion area, while MobileNetV3-Small exhibited a weaker localization, particularly in low-contrast images. These qualitative results are consistent with the quantitative performance metrics reported in Section 3.3.

#### 3.5.2. SHAP Analysis

**Figure 14:**
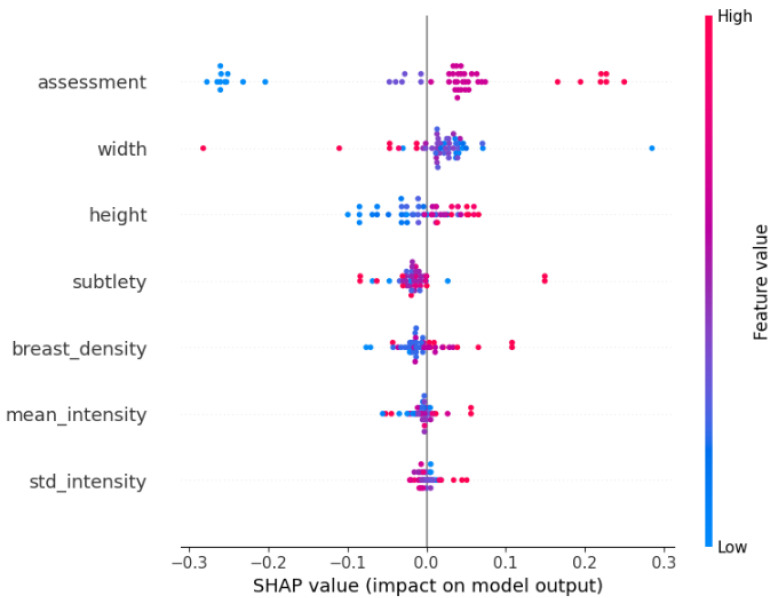
SHAP summary plot for the SVM model. Each point represents one case from the test set; its horizontal position shows the impact of the feature on the model output (negative values push the prediction towards benign, positive values towards malignant), and the colour encodes the feature value (red = high, blue = low).

**Figure 15:**
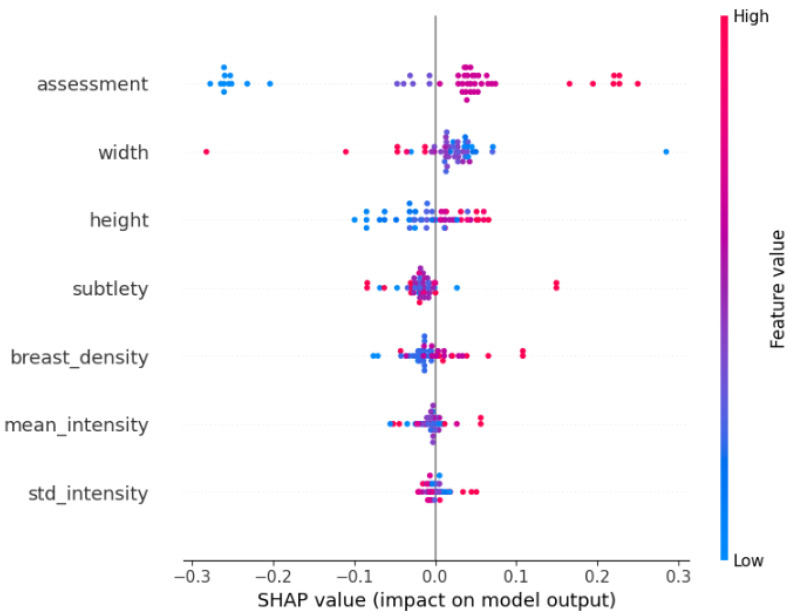
SHAP summary plot for the XGBoost model. Each point represents one case from the test set; the horizontal axis indicates the contribution to the malignant prediction, and the colour denotes the corresponding feature value (red for high values, blue for low values), highlighting which handcrafted variables most strongly drive malignant classifications.

For the SVM and XGBoost models, explainability was assessed using SHAP values [8], which quantify the contribution of each handcrafted feature to the final prediction. Figure 14 and  Figure 15 present the SHAP summary plots obtained from the test set.

In the SHAP summary plots, each dot corresponds to a mammogram; the horizontal coordinate represents whether the feature pushes the prediction towards the benign or malignant class, and the colour (red for high values, blue for low values) indicates the actual feature value for that case.

#### 3.5.3. Summary of the Interpretation

Together, the Grad-CAM and SHAP analyses confirm that the models’ decision processes were aligned with clinically relevant information. Deep-learning architectures identified spatially meaningful regions corresponding to lesions, while traditional classifiers weighted physically interpretable features. This complementarity between spatial and statistical explanations supports the overall validity of the modeling approach and enhances transparency for potential clinical applications.

### 3.6. Interactive Demonstration Interface

As part of the production-oriented validation of the proposed models, a lightweight interactive application was developed using Streamlit. The purpose of this tool is not to provide a full clinical interface, but to demonstrate how trained models can be consumed in a real-time setting.

The application allows users to upload mammography images in DICOM or PNG format. Once uploaded, the image is preprocessed through the same pipeline used during training and predictions are generated using the selected model. For convolutional networks, Grad-CAM visualizations are also computed and displayed to highlight the regions that contributed the most to the classification.

Although intentionally simple, this demonstrator illustrates how the experimental models can be exposed through an accessible interface and evaluated interactively. It also reflects a production-aware design consistent with Machine Learning Operations practices, showing how trained models can be packaged, served, and inspected as part of an end-to-end workflow. A visual summary of the Streamlit-based demonstration interface is included in the Supplementary Materials (Figures S1–S4).

### 3.7. Comparative Analysis

To contextualize the experimental results obtained in this study, the performance of the proposed models was compared with previously published works identified by systematic review conducted under the PRISMA 2020 statement protocol [11]. A total of 45 articles were analyzed, of which ten representative studies were selected for direct comparison due to their methodological similarity, reported metrics, and relevance to breast-cancer detection using medical imaging and artificial intelligence.

### 3.8. Overview of Related Work

Among the studies included through the PRISMA screening, convolutional neural networks (CNNs) represented the predominant approach to breast cancer detection, while XGBoost appeared as one of the most frequently used ensemble methods for tabular or feature-based classification. CNN-based works such as Arooj et al. [19], Aldhyani et al. [20] and Gürcan et al. [21] reported AUC-ROC values above 0.96, confirming the robustness of deep architectures in mammographic image datasets. Similarly, studies such as Aljuaid et al. [22], Hasan et al. [23], and Hejduk et al. [24] demonstrated accuracies greater than 0.90 using transfer learning and specialized CNN configurations. Other works, including Das et al. [25], and Sureshkumar et al. [26], achieved high performance in various imaging modalities such as histology and ultrasound, reflecting the generalizability of convolutional approaches in cancer detection tasks.

However, XGBoost-based methods also achieved competitive performance. Liang et al. [27] and Chen et al. [28] obtained AUC-ROC values close to 0.98 when applying feature engineering and optimization strategies. Zhu et al. [29] reached an AUC-ROC of 0.98 and perfect precision on mammographic data, while Jaddoa et al. [30] and Hoque et al. [31] reported accuracies above 0.94 using structured features extracted from clinical or imaging datasets. Although models such as Arora et al. [32] and Yurtseven et al. [33] achieved slightly lower recall values (0.71–0.80), they confirmed that ensemble methods remain effective alternatives when computational simplicity or explainability is prioritized.

### 3.9. Comparison with This Study

The CNN architectures developed in this work (ResNet-50, EfficientNetB0, and MobileNetV3-Small) achieved AUC-ROC values between 0.60 and 0.95, comparable to those reported in previous studies that used the same CBIS-DDSM [10] dataset. The XGBoost model based on handcrafted statistical features reached an AUC-ROC of 0.90, placing it within the same range as similar gradient-boosting approaches found in the literature. Overall, the results obtained are consistent with prior evidence suggesting that deep CNNs tend to outperform classical models when sufficient data and augmentation strategies are applied, while SVM and XGBoost remain effective on reduced or feature-engineered datasets.

### 3.10. Comparison Study for CNNs

Table 6 summarizes the main characteristics and performance metrics of the selected studies compared to the models implemented in this work.

### 3.11. Comparison Study of Classical Machine Learning Models

Table 7 summarizes the main characteristics and performance metrics of the selected studies compared to the XGBoost model implemented alongside the CNNs mentioned above.

## 4. Discussion

The models developed in this study were evaluated on a test subset that represents 20% of the total data. Given the clinical nature of the classification task, the main evaluation metric was Recall (Sensitivity), measure the ability of the model to correctly identify pathological cases. In medical contexts, minimizing false negatives is critical, since misclassifying a malignant case as benign can have severe consequences.

### 4.1. Limitations

The mammograms were resized so that full-field images were downsampled to 224 × 224 pixels, matching the input dimensions required by the pretrained CNN architectures used for transfer learning. This design choice enabled the reuse of ImageNet-trained backbones and substantially reduced computational cost, which was important to systematically compare several architectures and perform hyperparameter optimization. However, this resizing, while sufficient for global lesion-level classification, may reduce the model sensitivity to very small structures, such as microcalcification clusters. Previous studies have reported that lowering spatial resolution can impair the detection of small lesions and that multi-scale or patch-based approaches can improve the detection of both macro- and micro-level findings. In the present work, we did not perform an explicit ablation study on different input resolutions, so the quantitative impact of this design decision on microcalcification detection cannot be fully characterized. The reported global metrics (for example, an AUC-ROC of 0.95 for ResNet-50) should therefore be interpreted as overall diagnostic performance on CBIS-DDSM, rather than as a guaranty of optimal sensitivity for micro-lesions. Future work should explicitly evaluate per-lesion performance, particularly for microcalcifications, and compare single-scale and multi-scale or patch-based strategies under controlled conditions.

The architectures evaluated here are generic CNN backbones adapted to mammography, rather than models explicitly tailored and trained from scratch for breast imaging. Leveraging transfer learning was a deliberate decision to take advantage of large-scale pretrained weights and to obtain a robust baseline across multiple architectures in a common experimental setting. More specialized models operating at higher native resolutions or integrating view-wise information may ultimately achieve superior performance but would require considerably greater development effort, annotation resources, and computational budgets. Similarly, the present study focuses on a single curated public dataset (CBIS-DDSM), which provides a controlled and reproducible benchmark, but does not capture the full heterogeneity of acquisition devices and clinical populations. No external validation cohort was available, and we did not perform cross-dataset or multi-institutional testing, so the generalizability of the models beyond CBIS-DDSM remains uncertain. Rigorous external validation on independent datasets from different centers will be essential before any clinical deployment is considered.

From an experimental perspective, several additional limitations should be noted. First, statistical tests comparing model performance, such as *p*-values or confidence intervals for differences in AUC-ROC, were not computed, and the reported metrics should therefore be interpreted descriptively rather than as formally significant differences between architectures. Second, the classical machine learning models (XGBoost and SVM) were trained on a relatively compact set of clinical and morphological features. Although this choice reflects a realistic tabular representation and facilitates interpretability, it limits direct comparability with studies that use high-dimensional radiomic feature sets. Extending the current pipeline to incorporate radiomics would enable for a more direct comparison with that body of work. Finally, the risk-of-bias assessment presented in Section 2.6 was qualitative in nature. Although it provides an initial assessment of methodological quality, future studies could benefit from adopting structured instruments such as PROBAST [51] to perform a more systematic and rigorous evaluation of bias and applicability.

### 4.2. Format of Data and Impact of Training

The original mammograms in CBIS-DDSM are stored as DICOM files, which are well suited for clinical workflows but less convenient for large scale deep learning pipelines due to their metadata structure and input output overhead. To streamline preprocessing and training while maintaining compatibility with standard computer vision libraries, we converted the images to PNG format. In an initial implementation, mammograms were exported as 8 bit PNGs, which simplified handling but compressed the available dynamic range and led to a measurable decrease in validation performance compared with experiments that operated directly on the DICOM data.

To address this, the conversion pipeline was redesigned to produce 16 bit PNGs that preserve the original bit depth and gray level information. This adjustment recovered the lost performance while still providing the practical advantages of working with PNG files, such as faster disk access and simpler integration into the training code. As a result, preprocessing, cleaning, and training became more efficient without sacrificing the diagnostic content of the images. The final DICOM to PNG conversion script is included in the GitHub repository accompanying this work, allowing other researchers to reuse and adapt this step in their own pipelines.

### 4.3. Positioning with Respect to Related Work and Classical Models

When contrasted with the studies identified through the PRISMA 2020 systematic review, the performance of the models developed in this work falls within the range reported by recent CNN based approaches on mammography datasets (cf. Table 6 and Table 7). Several state of the art methods achieve slightly higher AUC-ROC values under task-specific or dataset-specific configurations, whereas the models evaluated here were trained and assessed under a unified experimental protocol on CBIS-DDSM. Rather than focusing exclusively on incremental gains in headline metrics, the present study deliberately emphasised the construction of a reproducible, end-to-end pipeline that spans data discovery, preprocessing, hyperparameter optimization, experiment tracking, and model deployment. Using a common MLOps workflow for all models allowed consistent hyperparameter tuning, logging, and model registration, thereby enabling fair comparison and facilitating downstream integration.

Importantly, the proposed pipeline was designed with deployment in mind. The same infrastructure that supports experimentation also produces ready-to-use artefacts, including a sample application that performs inference on mammograms and returns both class predictions and explainability outputs. In this way, the contribution of the study extends beyond individual model performance to provide a practical and extensible framework that can be adapted, scaled to additional datasets, or integrated into clinical decision-support systems in future work.

### 4.4. Explainability and Qualitative Analysis

Beyond quantitative metrics, qualitative analyses were performed to verify whether CNNs focused on clinically relevant regions. Using Grad-CAM visualizations, it was observed that ResNet-50 and MobileNetV3-Small consistently focused on breast tissue and lesion areas, while EfficientNetB0 was more sensitive to variations in input format, showing less localized activation in PNG images. These visual results suggest that the models learned to pay attention to diagnostically significant structures rather than noise or background artifacts. They provide qualitative insight into model attention and feature contributions.

For the classical machine-learning models (XGBoost and SVM), SHAP summary plots (Figure 14 and Figure 15) revealed that predictions were overwhelmingly driven by a small set of simple, globally computed image descriptors rather than by detailed radiological annotations. The features with the highest absolute SHAP values were the standard deviation of pixel intensity (std_intensity), mean pixel intensity (mean_intensity), height and width, assessment (BI-RADS) and subtlety. These intensity- and size-based statistics dominated the decision process in both models, whereas more specific pathological descriptors (e.g., calcification type, calcification distribution, mass shape, or mass margin) contributed only marginally or not at all.

This finding is consistent with the intentionally minimalist feature set we engineered for the classical baselines and highlights a key difference from deep CNNs: whereas ResNet-50 learns complex hierarchical patterns directly from raw pixels and focuses attention on the annotated lesion (Grad-CAM heatmaps in Figure 13), XGBoost and SVM rely heavily on global brightness and contrast cues that are easy to extract but less specific to subtle malignant patterns such as microcalcification clusters or architectural distortion. This explains the substantially lower sensitivity of the classical models (0.74 and 0.66 versus 0.89 for ResNet-50) and underscores the advantage of end-to-end deep learning when detailed lesion morphology is critical for accurate diagnosis.

### 4.5. MLOps and Deployment-Oriented Workflows

Beyond predictive performance, clinical AI systems must be supported by workflows that ensure traceability and operational robustness. On our experiment, every training run and optimization trial is recorded together with its hyperparameters, metrics, artefacts, and resource usage, creating an auditable history of how each model version was produced. This systematic logging, combined with a central model registry, facilitates controlled roll-out of new versions, rollback if necessary, and reproducible re-training when new data become available. Such capabilities are directly relevant for future integration into regulated healthcare environments, where governance, monitoring, and documentation are as important as raw accuracy.

### 4.6. Toward Responsible Artificial Intelligence

Beyond predictive performance, the development of clinical AI systems must be guided by principles of responsible artificial intelligence, including transparency, traceability, and attention to potential sources of bias. In this study, we incorporated several practices aligned with these principles: model explainability was addressed through Grad-CAM for CNNs and SHAP analyses for XGBoost and SVM, reproducibility was strengthened by documenting and releasing the code and configuration used in the experiments, and auditability was supported through systematic logging of data versions, hyperparameters, metrics, and model artefacts. These elements do not, by themselves, resolve all ethical and fairness challenges, but they constitute practical steps toward making the behaviour of the models more interpretable and their lifecycle more traceable. Such properties are essential prerequisites for clinical deployment, where clinicians must be able to scrutinise predictions, regulators require clear documentation of model provenance, and future updates or adaptations need to be performed under controlled, auditable conditions.

## 5. Conclusions

This study applied artificial intelligence techniques to breast cancer diagnosis using mammographic images and associated clinical variables within an explainable and reproducible framework. Beyond evaluating individual models, the work focused on constructing a practical pipeline that can serve as a foundation for future clinical AI systems in this domain.

A functional and scalable workflow was developed to preprocess and analyse both DICOM images and structured clinical data. The system covered the full path from data standardization and quality control to model training, evaluation, and prediction generation, and was designed to support future extensions. Handling imaging and tabular information in parallel required addressing issues such as variable image quality, metadata cleaning, and reliable linkage between modalities at the patient and exam level.

Multiple models were trained and evaluated, including deep learning architectures (ResNet-50, EfficientNetB0, MobileNetV3-Small) and classical algorithms (XGBoost, SVM). This diversity enabled not only the comparison of convolutional networks on pixel-level inputs, but also the assessment of traditional methods when applied to curated clinical and morphological features. The best CNN (ResNet-50) achieved an AUC-ROC of 0.95 with a Recall of 89%, while the XGBoost model reached an AUC-ROC of 0.90 with a Recall of 74%, supporting the potential diagnostic value of both paradigms and their complementary role as decision support components.

Interpretability was a central aspect of the project. Grad-CAM visualizations showed that CNNs tended to focus on clinically relevant breast regions and lesion areas, while SHAP analyses for XGBoost and SVM clarified the influence of key variables such as BI-RADS assessment, breast density, and calcification characteristics. Taken together, these techniques suggest that the models based their decisions on medically meaningful patterns rather than purely spurious correlations, and provide a basis for qualitative inspection by clinicians.

A prototype application was implemented using Streamlit, allowing users to upload mammograms, select models, run inference, and visualize explainability outputs. This tool enables interactive exploration of model behaviour by researchers and clinicians and represents an initial step toward the integration of similar systems into radiological workflows.

Finally, throughout the project, principles of responsible artificial intelligence were considered, with particular emphasis on transparency, traceability, and explainability. The PRISMA-2020 checklists and additional screenshots supporting the reproducibility of this study are available in the Supplementary Materials (Table S1–S2 and Figures S1–S4, respectively). Code, configuration, and model artefacts were documented and versioned, and post hoc interpretation methods were systematically applied to illuminate the decision processes of both image-based and tabular models. While further work is needed to address fairness and regulatory aspects in a comprehensive manner, the pipeline developed here illustrates how technical performance, interpretability, and operational readiness can be jointly pursued when designing AI systems intended for real-world medical settings.

### Recommendations

Future research should:

- Promote the publication of anonymized clinical datasets for research, ensuring both data quality and compliance with privacy regulations.
- Establish standardized criteria for clinical metadata structure and documentation to improve interoperability and reproducibility between studies.
- Encourage clinical experts to validate the output of AI models to ensure medical coherence and alignment with professional practice.

## Figures and Tables

**Table 1:** Hyperparameters optimized with Optuna for the three CNN classifiers.

Hyperparameter	ResNet-50	EfficientNetB0	MobileNetV3-Small
Learning rate (backbone)	$6.41\times 10^{-5}$	$6.41\times 10^{-5}$	$5.0\times 10^{-5}$
Learning rate (classifier head)	$6.01\times 10^{-5}$	$6.01\times 10^{-5}$	$5.0\times 10^{-4}$
weight_decay	$8.68\times 10^{-5}$	$8.68\times 10^{-5}$	$5.0\times 10^{-4}$
gamma	1.1121	1.9349	2.0
dropout_rate	0.697	0.697	0.70
batch_size	64	64	64
early_stop_patience	16	16	10
alpha (class weights)	[1.0, 2.86, 1.0]	[1.0, 2.86, 1.0]	[1.0, 2.0, 1.0]

**Table 2:** Summary of results for CNN models.

Model	Recall	Accuracy	AUC-ROC
ResNet-50	89%	90%	0.95
EfficientNetB0	40%	40%	0.60
MobileNetV3-Small	43%	42%	0.60

**Table 3:** Hyperparameters optimized with Optuna for the XGBoost classifier.

Hyperparameter	Value
learning_rate	0.017283
max_depth	8
min_child_weight	2.673682
subsample	0.858516
colsample_bytree	0.784514
gamma	0.071834
reg_alpha	0.005930
reg_lambda	0.329732
n_estimators	659

**Table 4:** Hyperparameters optimized with Optuna for the SVM classifier.

Hyperparameter	Value
C	0.561151
kernel	linear
gamma	scale
class_weight	equal

**Table 5:** Summary of results for Machine Learning models.

Model	AUC-ROC	Recall	Accuracy
XGBoost	0.90	74%	72%
SVM	0.84	66%	63%

**Table 6:** CNN-based studies from the PRISMA selection and this study; missing values shown as —.

Study	Model Type	Dataset Used	AUC-ROC	Recall	Accuracy	F1	Precision
[19]	AlexNet	Mammography *	—	—	99.4%	—	—
[25]	ResNet-50	CBIS-DDSM, INBreast	—	83.2%	84.6%	83.2%	—
[22]	ResNet18	BreakHis	—	—	97.81%	—	—
[26]	Custom CNN	MIAS	—	—	86%	—	—
[20]	Custom CNN	BreakHis, BreCaHAD	—	97.99%	99.61%	99.95%	99.27%
[34]	DarkNet-53 + Cubic SVM	BUSI	—	—	99.1%	—	—
[21]	CNN + stacking ensemble	WBCD	0.9742	98.6%	97.72%	98.19%	97.87%
[35]	Custom CNN + stacking ensemble	MRI *	—	92%	—	—	95%
[23]	CNN	Mammography	0.895	—	90%	—	—
[24]	Custom CNN	ABUS	0.910	90.9%	90.9%	—	90.9%
[36]	CNN + extended ensemble	INBreast, CBIS-DDSM	0.94	90.2%	89.5%	—	—
[37]	ResNet101	MIAS, CBIS-DDSM	—	98.76%	98.63%	—	—
[38]	DenseNet-121	CBIS-DDSM	0.809	71%	73%	—	—
[39]	ViT + U-KAN	BreastDM, BUSI, MIAS, BreakHis, DDSM	—	98.3%	99.3%	—	—
[40]	EfficientNetB0	Mammography	—	96.55%	95.04%	96.00%	—
[41]	ResNet-50	MIAS	0.99	98.86%	99.24%	98.49%	98.13%
**Ours**	ResNet-50	CBIS-DDSM	**0.95**	**89%**	**90%**	**87%**	**86%**
**Ours**	EfficientNetB0	CBIS-DDSM	**0.60**	**40%**	**40%**	**35%**	**38%**
**Ours**	MobileNetV3-Small	CBIS-DDSM	**0.60**	**43%**	**42%**	**35%**	**42%**

Note. An asterisk (*) indicates that for studies based on private datasets, only the imaging modality (for example, mammography, ultrasound, histology) is reported instead of a dataset name. Bold captions are used to identify the results obtained during our experimentation.

**Table 7:** XGBoost and SVM-based studies from the PRISMA selection and this study; missing values shown as —.

Study	Model Type	Dataset Used	AUC-ROC	Recall	Accuracy	F1	Precision
[33]	XGBoost	Microwaves *	—	80%	81%	—	—
[32]	XGBoost	Breast Cancer RSNA	—	86%	89%	86%	86%
[42]	XGBoost	Ultrasound *	0.857	—	—	—	—
[30]	XGBoost	Coimbra	—	97%	98.32%	99%	98%
[27]	XGBoost	Mammography *	0.89	88.45%	90.24%	89.52%	90%
[29]	LightGBM-PSO	Wisconsin Breast Cancer Diagnosis (WBCD)	0.9870	97.4%	99%	98.68%	100%
[28]	XGBoost+SMA	Mammography	—	—	98.45%	98.47%	—
[31]	XGBoost	WBCD	—	95.24%	94.74%	—	90.91%
[43]	XGBoost	Ultrasound *	0.842	—	—	—	—
[44]	LightGBM	CBIS-DDSM and CMMD	—	99.3%	99.5%	—	99.5%
[45]	XGBoost	MRI *	0.940	84%	88%	—	—
[46]	LGBM	CMMD	—	80%	81%	77%	77%
[47]	XGBoost	Breast Cancer Prediction (Kaggle)	0.926	87.0%	87.1%	87.9%	89.0%
**Ours**	XGBoost	CBIS-DDSM	**0.90**	**74%**	**73%**	**74%**	**73%**
[43]	SVM	Ultrasound *	0.904	—	—	—	—
[48]	pdMISVM	BreaKHis	—	94.2%	89.1%	92.5%	92.3%
[49]	SVM	WBCD	—	—	99.12%	—	—
[50]	IQI-BGWO-ISVM (Optimized SVM-RBF, 10-CV)	MIAS	—	98.9%	99.2%	—	—
[47]	SVC	Breast Cancer Prediction (Kaggle)	0.978	88.0%	88.71%	89.0%	91.0%
**Ours**	SVM	CBIS-DDSM	**0.84**	**66%**	**84%**	**63%**	**63%**

Note. An asterisk (*) indicates that for studies based on private datasets, only the imaging modality (for example, mammography, ultrasound, histology) is reported instead of a dataset name. Bold captions are used to identify the results obtained during our experimentation.

## Data Availability

The datasets used in this study are publicly available. The CBIS-DDSM dataset can be accessed at https://www.cancerimagingarchive.net/collections/cbis-ddsm/ (accessed on 4 December 2025). No new datasets were created in this study.

## Supplementary Materials

The following supporting information can be downloaded at: https://www.mdpi.com/article/10.3390/medicina61122237/s1, Table S1: PRISMA 2020 main checklist; Table S2: PRISMA 2020 abstract checklist; and Figures S1–S4: screenshots of the Streamlit developed application demonstrating the multimodal screening tool.
